# Stigma towards women requesting abortion and association with health facility staff facilitation and obstruction of abortion care in South Africa

**DOI:** 10.3389/fgwh.2023.1142638

**Published:** 2023-06-15

**Authors:** Abongile Jim, Makgoale Magwentshu, Jamie Menzel, Stephanie Andrea Küng, Sa-Asa August, Justine van Rooyen, Rumbidzayi Chingwende, Erin Pearson

**Affiliations:** ^1^ Ipas South Africa, Johannesburg, South Africa; ^2^ Ipas, Chapel Hill, NC, United States

**Keywords:** abortion (attitudes toward), stigma and discrimination, South Africa, induced abortion, provider attitudes

## Abstract

**Background:**

Abortion stigma has been shown to influence provider attitudes around abortion and may decrease provider willingness to participate in abortion care, or lead some to obstruct care. However, this link remains understudied.

**Methods:**

The present study uses baseline data collected through a cluster-randomized controlled trial in 16 public sector health facilities in South Africa in 2020. A total of 279 clinical and non-clinical health facility workers were surveyed. Primary outcome measures included: 1) willingness to facilitate abortion care in eight hypothetical scenarios, 2) facilitation of abortion care in the last 30 days, and 3) obstruction of abortion care in the last 30 days. Logistic regression models were used to assess the association between level of stigma as measured through the Stigmatizing Attitudes, Beliefs and Actions Scale (SABAS) and the primary outcomes.

**Results:**

Overall, 50% of respondents in the sample were willing to facilitate abortion care in each of the eight scenarios, with differences in willingness based on the abortion client's age and personal situation in each scenario. Over 90% reported facilitating abortion care in the last 30 days, but 31% also reported having obstructed abortion care in the last 30 days. Stigma was significantly associated with willingness to facilitate abortion care and actual obstruction of abortion care in the last 30 days. Controlling for covariates, odds of willingness to facilitate abortion care in every scenario decreased with every one-point increase in SABAS score (reflecting more stigmatizing attitudes), and odds of obstructing abortion care increased with every one-point increase in SABAS score.

**Conclusions:**

Lower abortion stigma on the part of health facility workers was associated with willingness to facilitate abortion access but not actual facilitation of abortion services. Higher abortion stigma was associated with actual obstruction of an abortion service in the last 30 days. Interventions to reduce stigma towards women seeking abortion, and particularly negative stereotyping, among *all* health facility staff is key to ensuring equitable and non-discriminatory access to abortion.

**Trial registration:**

Retrospectively registered on clinicaltrials.gov (ID: NCT04290832) on February 27, 2020.

**Plain english summary:**

The link between stigma against women seeking abortion and decisions around whether to provide, abstain, or obstruct abortion care remains understudied. This paper assesses how stigmatizing beliefs and attitudes towards women seeking abortion in South Africa affects willingness to facilitate abortion care and actual facilitation or obstruction of abortion care in practice. A total of 279 clinical and non-clinical health facility workers were surveyed between February and March 2020. Overall, half of respondents in the sample were willing to facilitate abortion care in each of the eight scenarios, with important differences in willingness by scenario. Almost all respondents reported facilitating an abortion procedure in the last 30 days, but one in three also reported having obstructed abortion care in the last 30 days. More stigmatizing attitudes corresponded to decreased willingness to provide abortion care and increased odds of obstructing abortion care. Results show that stigmatizing attitudes, beliefs, and actions toward women who seek abortion shape how clinical and non-clinical staff in South Africa feel about their participation in abortion services and whether they obstruct this care. Facility staff hold great power in determining whose abortions are facilitated and whose are obstructed, resulting in stigma and discrimination being openly perpetuated. Continuous work to reduce stigma towards women seeking abortion among *all* health workers is key to ensuring equitable and non-discriminatory access to abortion for all.

## Background

The South Africa abortion law reform in 1996 transformed the landscape of abortion access in the country. South Africa's Choice on Termination of Pregnancy (CTOP) Act is among the most liberal abortion laws in the world, allowing abortion on request up to 12 weeks of pregnancy and up to 20 weeks in certain circumstances including socioeconomic reasons (1, 2). After 20 weeks, abortion is still legal if continuation of the pregnancy presents a risk to the person's health or severe handicap to the fetus. The CTOP Act explicitly states that minors are not required to notify a parent or guardian, nor are rape survivors required to provide any documentation of the assault in order to receive an abortion (3). In addition, the CTOP Act makes it illegal for anyone to prevent a lawful termination of pregnancy (TOP) service (4).

Despite this liberal law, safe facility-based abortion remains out of reach for many people in South Africa; it is estimated that over 50% of abortions in the country occur outside of health facilities, often under unsafe circumstances (5, 6). Many factors may determine access to facility-based abortion care, and these operate both on the demand side (financial barriers, distance to hospitals or clinics, lack of childcare or time off from work or school, fear of disclosure, perceived or experienced stigma or mistreatment within medical settings) and the supply side (legal restrictions, lack of supplies, lack of willing or trained staff). A wealth of literature on abortion attitudes points to pervasive abortion stigma in South Africa and beyond, among healthcare workers and society at large (7–10). On the demand side, stigma may impact abortion access by preventing women^1^ from seeking abortions in the first place or may lead people to avoid health facilities in favor of traditional medicine or self-managed abortion (11, 12). On the supply side, qualitative literature on conscientious objection (CO) identifies experienced stigma from society and colleagues *and* stigma against people who get abortions as two principal reasons why healthcare providers object. According to these studies, providers and other staff may refuse to provide abortion for fear of or experiences of exclusion, condemnation, or violence from other providers, families, friends, and communities (13, 14). Clinicians in South Africa who do provide abortion have detailed experiences of harassment, from being called “baby killers” to having their cars stoned (15). CO literature has also documented stigma from healthcare workers against women who access abortion, seeing these women as irresponsible, or their abortions as preventable, especially women seeking abortion for reasons other than rape, incest, or life endangerment (16, 17).

However, literature on provider attitudes toward abortion and CO has also made clear the nuance in decisions around the provision, refusal, or obstruction of abortion care, with many providers choosing to participate only in certain situations or settings (14, 16–18). While we know providers may cite perceived, experienced, or enacted stigma as reasons to not participate in abortion care, we do not know the extent of this problem. There is a lack of quantitative research that demonstrates the association between stigma and willingness to facilitate access to abortion care among health facility staff, and whether this willingness translates into actual facilitation or obstruction of abortion care. An evaluation of Provider Share Workshops in sub-Saharan Africa found significant relationships between provider experiences of stigma and stigmatizing attitudes toward women seeking abortion, but this study did not assess whether such attitudes determined provision or non-provision of abortion care (19). Additionally, one study from Ethiopia found that stigmatizing attitudes and beliefs among midwives was marginally but significantly associated with willingness to provide abortion (20). However, evidence also suggests that abortion stigma may not always translate into refusal or obstruction of abortion care in practice, as some health care workers view their personal attitudes as entirely separate from their clinical duties (23).

Our study aims to explore, quantitatively, how stigmatizing attitudes, beliefs, and actions *toward women seeking abortions* contributes to decisions around participation in abortion care. To our knowledge, no literature exists that examines quantitatively how stigma towards women seeking abortion care may affect health facility staff participation in abortion care in South Africa. Specifically, we assess how stigmatizing beliefs and attitudes towards women receiving abortion affects willingness to facilitate access to abortion care in theory and how these attitudes affect practice, including facilitation or obstruction of abortion services among clinical and non-clinical hospital and health center staff in two provinces in South Africa.

## Methods

The present study uses baseline data collected through a cluster-randomized controlled trial (clinicaltrials.gov ID: NCT04290832). The trial was designed to evaluate the effect of an intervention focused on mitigating the impact of CO on women's reproductive health in South Africa and Mexico, but the study could not be completed as planned due to the COVID-19 pandemic. The present analysis focuses on baseline data collected in South Africa before the pandemic began. The sampling frame for the study included 58 facilities in Gauteng and Limpopo provinces where Ipas South Africa, a non-governmental organization (NGO) focused on advancing access to sexual and reproductive health services including safe abortion, had trained at least one abortion provider. Baseline data collection was completed in 16 of the 26 study sites randomly assigned to the first round of data collection before the study was halted due to the COVID-19 pandemic in March 2020. After the first round of data collection, the intervention was to be refined and then tested in the remaining 32 facilities. The 16 study sites where baseline data were collected do not differ meaningfully from the 10 sites that did not complete data collection before the halt nor any of the sites assigned to the second round of data collection.

Staff lists were obtained from all selected facilities, and staff were stratified into four groups for sampling: (1) doctors and nurses who provide abortion, (2) doctors who do not provide abortion, (3) nurses who do not provide abortion, and (4) support staff (including health promoters, clerks, cleaners, security guards, and porters). The present analysis only includes doctors and nurses who do not provide abortion and support staff, as stigma scales were not applied among clinicians who provide abortion. Clinical staff who do not provide abortion may do so for several reasons, including working in other units (maternity wards, primary care, etc.), lack of training in abortion, or lack of interest or objection to abortion. Within the nurse and support staff strata, facility administrators provided information on level of engagement with abortion clients, ranging from “high engagement” (e.g., staff working in or near the abortion ward, such as in the labor and delivery ward) to “low engagement” (e.g., staff working in wards physically distant from the abortion ward who rarely engaged with abortion clients). Within the nurse stratum, only those with “high engagement” were retained in the sampling frame, and within the support staff stratum those with “high engagement” or “medium engagement” were retained in the sampling frame. Within each facility, eight people were sampled in each of the three strata (doctors, nurses, and support staff) using simple random sampling without replacement. This study received ethics approval from The Human Sciences Research Council's (HSRC) Research Ethics Committee in South Africa (Protocol #: REC 9/21/11/18) and all respondents gave their informed consent to participate.

A total of 279 clinical (including general practitioners and specialists, nurses, and midwives who do not provide abortion) and non-clinical (admin or support staff) health facility workers were surveyed between February and March 2020. This number does not include the 68 health facility staff who were included in the sampling frame but who did not complete baseline surveys for a variety of reasons. Trained interviewers including three Ipas staff members and five consultants who administered the baseline survey in-person using CommCare software. Surveys took approximately 45 min to complete.

We used three binary outcome measures: (1) willingness to facilitate abortion care in eight hypothetical scenarios, (2) facilitation of abortion care in the last 30 days, and (3) obstruction of abortion care in the last 30 days. For the first outcome measure, respondents were asked how willing they would be to facilitate abortion care in eight different scenarios. The eight scenarios were developed by reproductive rights experts and physicians at Ipas to reflect scenarios where providers are known to be more or less likely to employ CO, including rape, financial/economic reasons, mental health, physical health, congenital defects, and lack of contraception (Table 1). Respondents could answer that they were fully willing to facilitate the service, somewhat willing, or not willing. Fully willing and somewhat willing were collapsed for the purposes of analysis due to the small number of somewhat willing responses.

**Table 1:** Outcome measures.

Outcomes	Measures
Scenarios	**Scenario 1**: A 13-year-old rape survivor requests termination of pregnancy (TOP). She has a gestation of 23 weeks.
	**Scenario 2**: A 34-year-old woman living in poverty with her three children and her partner is not supporting her financially. She shares that she suffers from deep depression and requests TOP. She has a gestation of 18 weeks.
	**Scenario 3**: A 26-year-old woman has received a diagnosis of cervical cancer. She has a gestation of 16 weeks and her oncologist recommends a TOP which she agrees with.
	**Scenario 4**: A 17-year-old woman who is in her second year of nursing studies requests TOP. She says that her parents will not support her to complete her studies if they find out she is pregnant.
	**Scenario 5**: A 39-year-old woman requests TOP. She explains that she lives in a small apartment and is employed as a domestic worker. She will not be able to keep her job if her employer finds out that she is pregnant.
	**Scenario 6**: A 23-year-old woman requests TOP after learning that her fetus has serious congenital defects. She says that she feels desperate and terrified about the idea of giving birth to a fetus that her gynecologist says will not survive
	**Scenario 7**: A 29-year-old woman says that a “friend” had forced her to have sex a couple of weeks ago. She has not reported this to the police and says, “I just don’t want to think about that night.”
	**Scenario 8**: A 31-year-old woman requests TOP after learning she is pregnant. She says that she has been using condoms with her boyfriend, but she had forgotten to use one a couple of weeks ago.
Facilitation of Abortion Care, last 30 days	**YES to one of the following:**
	Helped a woman find the TOP unit, e.g. provided directions or escorted her;
	Provided positive support to a woman seeking TOP care;
	Gave accurate information to a woman seeking TOP care
Obstruction of Abortion Care, last 30 days	**YES to one of the following:**
	Would not provide or gave incorrect directions to the TOP unit when asked by a woman seeking TOP care;
	Told a woman seeking TOP care that TOP services are not provided at this facility;
	Referred a woman seeking TOP care to another facility so I would receive an incentive;
	Tried to convince a woman seeking TOP care that she should not have TOP;
	Called a woman names or spoke with her harshly for seeking TOP care;
	Told a woman seeking TOP care that she cannot receive services because she doesn’t have proof of address;
	Told a woman seeking TOP she is too young or she must come with a parent /guardian;
	Told a woman seeking TOP she must come with her husband/partner

Respondents were also asked a series of yes or no questions to gauge facilitation or obstruction of abortion care in the last 30 days (Table 1). If the respondent reported helping a person find the abortion ward, providing positive support to a person seeking abortion care, *or* giving accurate information to a person seeking abortion care, they were identified as having facilitated access to abortion care in the last 30 days. If the respondent reported not providing or giving incorrect directions to the abortion ward, telling a woman that abortion services were not provided at the facility, referring a woman elsewhere for abortion to receive an incentive, convincing a woman that she should not have an abortion, calling a woman names or speaking to her harshly for seeking an abortion, *or* requesting additional information or accompaniment (proof of address, husband/partner, or parent/guardian), they were categorized as having obstructed abortion care in the last 30 days.

The key independent variable was level of stigma as measured through the Stigmatizing Attitudes, Beliefs and Actions Scale (SABAS). SABAS is a tool specifically designed to measure abortion stigma at the individual and community level (21). Respondents are asked how much they agree with a series of 18 statements on a 5-point Likert scale ranging from strongly disagree to strongly agree. Some statements that compose SABAS include, “A woman who has an abortion cannot be trusted,” “A woman who has an abortion is a bad mother,” “I would stop being friends with someone if I found out that she had an abortion,” and “A woman who has an abortion can make other people fall ill or get sick”. The full 18-item SABAS instrument contains three subscales: negative stereotyping (8 items), exclusion and discrimination (7 items), and fear of contagion (3 items). Each statement was scored from 1 to 5, with higher scores reflecting more stigmatizing attitudes; potential SABAS scores range from 18 to 90 for the full scale, 8–40 for the negative stereotyping subscale, 7–35 for the exclusion and discrimination subscale, and 3–15 for the fear of contagion subscale (see Additional file 1 for SABAS scale items). SABAS was initially developed and validated for use at the community level in Ghana and Zambia (21), and it was subsequently validated with midwives in Ethiopia (20). Holcombe et al. found that one sub-scale, fear of contagion, had limited face validity, and recommended an adapted version for health workers, which has not yet been developed (20). The SABAS has not been used with other types of facility staff.

We first ran descriptive statistics and bivariate tests to assess associations between stigma and the outcomes of interest. Then, for each outcome (the eight abortion scenarios, facilitation of abortion care in the last 30 days, and obstruction of abortion care in the last 30 days), we ran multivariable logistic regression models to assess the relationship between the SABAS stigma score and outcomes. We controlled for socio-demographic characteristics, including age, province, sex, religion, ethnicity, marital status, and length of time working at the health facility. As SABAS scores were significantly different by clinical vs. non-clinical health facility staff, we also controlled for position at facility in our models. Religion was dichotomized into Christian vs. non-Christian to account for the small number of respondents who identified with religions other than Christian. To assist with analysis, ethnicity was also collapsed into Black South African vs. non-Black South African (Colored, Indian, and Another Ethnicity). Marital status was collapsed into three categories: currently married/living with partner, separated/divorced/widowed, and never married.

We performed several sensitivity analyses. We ran bivariate analyses for association between SABAS score and each question about facilitation and obstruction that were used to build the overall facilitation or obstruction outcomes. SABAS scores were run in the models as a combined score and as separate subscales to assess which domains were driving significance. We also hypothesized possible interactions between Christianity and stigma given literature showing the interplay between religion and stigma (7, 22); interaction terms were not significantly associated with any outcomes and were not retained in the final models.

## Results

Characteristics of the 279 clinical and non-clinical surveyed staff who do not provide abortion services are presented in Table 2. A slight majority (54%) of respondents are admin/support staff and more than half (54%) reside in Gauteng. Over three-quarters (80%) of respondents were female, and most (93%) self-identified as Christian. Ethnicity was varied among respondents, nearly one-quarter (23%) identified as Tsonga, followed by Venda (15%) and Sotho (15%), Zulu (13%), and Pedi (10%). Nearly half of respondents were either married or living with their partners (49%), and more than one-third (36%) were unmarried. Most respondents (72%) had worked at their facilities for over five years. The average SABAS score among all respondents was 39.3 (range: 18–66), representing medium to low levels of stigma among our sample. Socio-demographic characteristics were not significantly different between clinical and non-clinical staff. Clinical staff on average worked fewer years at the facility than non-clinical staff. Clinical staff also had lower SABAS scores compared to non-clinical staff, a mean score of 36.4 compared to 41.6 (*p* < 0.001), indicating less stigmatizing attitudes among clinical staff.

**Table 2:** Characteristics of the sample (*n* = 279).

Characteristic	Clinical Staff		Support Staff		Total		*p*-value
	*n* = 127		*n* = 152		*n* = 279		
	*n*	(%)	*n*	(%)	*n*	(%)	
Region							0.597
Gauteng	67	(53)	85	(56)	152	(54)	
Limpopo	60	(47)	67	(44)	127	(46)	
Age							0.639
<35	31	(24)	30	(20)	61	(22)	
35 to 49	55	(43)	69	(45)	124	(44)	
>50	41	(32)	53	(35)	94	(34)	
Sex							0.198
Female	107	(84)	116	(76)	223	(80)	
Male	20	(16)	35	(23)	55	(20)	
Religion							
Islam	3	(2)	0	(0)	3	(1)	0.057
Hinduism	2	(2)	0	(0)	2	(1)	0.120
Christianity	119	(94)	140	(92)	259	(93)	0.607
Tradition/African/Ancestors	2	(2)	6	(4)	8	(3)	0.237
None	1	(1)	3	(2)	4	(1)	0.406
Don’t know	0	(0)	1	(1)	1	(0)	0.360
Refused	0	(0)	3	(2)	3	(1)	0.111
Ethnicity							0.131
Colored	3	(2)	4	(3)	7	(3)	
Indian	4	(3)	0	(0)	4	(1)	
Ndebele	6	(6)	3	(2)	9	(3)	
Swati	1	(1)	2	(1)	3	(1)	
Tsonga	27	(21)	37	(24)	64	(23)	
Tswana	12	(9)	16	(11)	28	(10)	
Venda	22	(17)	21	(14)	43	(15)	
Xhosa	4	(3)	6	(4)	10	(4)	
Zulu	14	(11)	21	(14)	35	(13)	
Sotho	16	(13)	26	(17)	42	(15)	
Pedi	12	(9)	16	(11)	28	(10)	
Another ethnicity	6	(5)	0	(0)	6	(2)	
Marital Status							0.491
Currently married	56	(44)	54	(36)	110	(39)	
Separated	3	(2)	3	(2)	6	(2)	
Divorced	9	(7)	11	(7)	20	(7)	
Widowed	6	(5)	8	(5)	14	(5)	
Living with partner	15	(12)	12	(8)	27	(10)	
Never married	37	(29)	63	(41)	100	(36)	
Refused	1	(1)	1	(1)	2	(1)	
Time at facility (years)							0.019*
<5 years	44	(35)	33	(22)	77	(28)	
5–10 years	45	(35)	52	(34)	97	(35)	
>10 years	38	(30)	67	(44)	105	(38)	
Average total SABAS score (range 18–90)	36.4		41.6		39.3		<0.001*

*p < 0.05.

**Figure 1:**
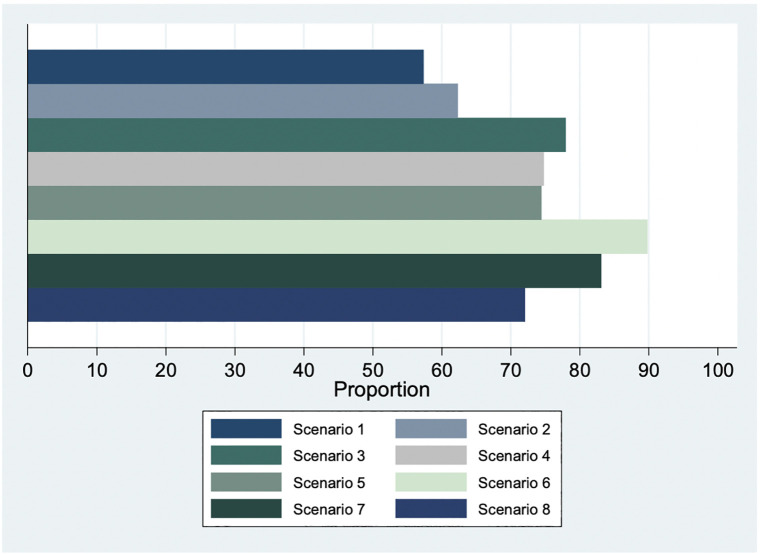
Willingness to facilitate abortion care, eight scenarios (*n* = 279).

Overall, more than half of respondents in the sample stated they were willing to facilitate abortion care in each of the 8 hypothetical scenarios, across clinical and non-clinical staff (Figure 1). The scenario for which fewest respondents were willing to facilitate abortion care was Scenario 1 (a 13-year-old rape survivor with a gestation of 23 weeks; 57% willing to facilitate). The scenario that received the widest support in willingness to facilitate was Scenario 6 (A 23-year-old woman requests TOP after learning that her fetus has serious congenital defects; 90% willing to facilitate).

**Figure 2:**
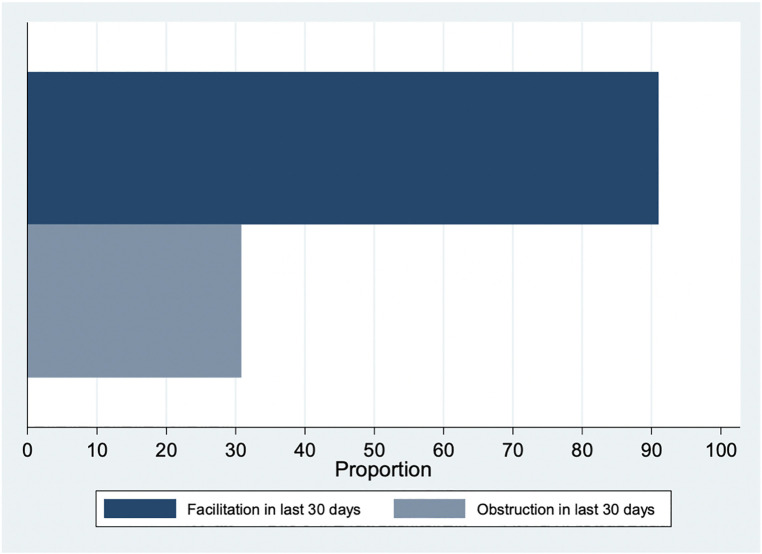
Facilitation and obstruction of abortion care in last 30 days (*n* = 279).

A large majority of respondents (91%) had done something to facilitate access to abortion care in the last 30 days (93% among non-clinical staff and 88% among clinical staff; Figure 2). Of the three questions consolidated into our facilitation variable, the most frequent type of facilitation was helping a woman find the abortion unit (86%) followed by provision of accurate information (78%) and positive support (78%). In contrast, one in three respondents (31%) reported having done something to obstruct access to abortion in the last 30 days (34% among non-clinical staff and 27% among clinical staff; Figure 2). Of the eight questions consolidated into our obstruction variable, the most frequent type of obstruction was trying to convince a woman that she should not have an abortion (20%), followed by telling the woman she is too young or that she must come with a parent/guardian (12%).

In bivariate analyses, SABAS score was inversely associated with all eight “willingness to facilitate access” scenarios. Each scenario was significantly associated with SABAS score with the exception of Scenario 2 (A 34-year-old woman living in poverty and suffering from deep depression; *p* = 0.074). For all other scenarios, for every one-point increase in SABAS score, odds of willingness to facilitate the abortion service decreased significantly (Scenario 1: OR 0.96, *p* = 0.004; Scenario 3: OR 0.97, *p* = 0.04; Scenario 4: OR 0.96, *p* = 0.006; Scenario 5: OR 0.95, *p* = 0.003; Scenario 6: OR 0.93, *p* = 0.003; Scenario 7: OR 0.95, *p* = 0.007; Scenario 8: OR 0.94, *p* < .001). Controlling for covariates, there was a significant, negative association between SABAS score and willingness to facilitate access to abortion care in every scenario, including Scenario 2. Odds of willingness to facilitate abortion care in every scenario decreased with every one-point increase in SABAS score (Tables 3A, B). No other variables were significantly associated with willingness to facilitate across all scenarios. For Scenarios 2, 4, 5, and 8, clinical staff were significantly less likely to be willing to facilitate abortion care when compared to non-clinical staff, controlling for covariates.

**Table 3A:** Association of SABAS score and willingness to facilitate abortion care in scenarios 1–4 (*n* = 279).

	Scenario 1		Scenario 2		Scenario 3		Scenario 4	
	aOR	95% CI	aOR	95% CI	aOR	95% CI	aOR	95% CI
SABAS score	0.96*	[0.925–0.987]	0.96*	[0.929–0.992]	0.95*	[0.915–0.990]	0.93*	[0.899–0.972]
Female (ref male)	0.81	[0.405–1.605]	1.12	[0.552–2.257]	0.87	[0.385–1.990]	1.34	[0.601–2.988]
Age (ref <35)								
35–49	0.92	[0.446–1.895]	0.6	[0.280–1.281]	1.25	[0.517–3.002]	0.23*	[0.081–0.646]
50+	0.98	[0.400–2.391]	0.78	[0.306–1.996]	2.03	[0.677–6.091]	0.28*	[0.084–0.943]
Guateng (ref Limpopo)	1.67	[0.987–2.810]	1.38	[0.803–2.366]	1.65	[0.884–3.094]	0.82	[0.439–1.521]
Marital Status (ref Single)								
Married/living with partner	0.99	[0.549–1.779]	1.34	[0.730–2.456]	0.95	[0.474–1.900]	1.04	[0.517–2.084]
Divorced/Widowed/Separated	1.05	[0.454–2.430]	1.38	[0.581–3.277]	1.28	[0.466–3.537]	2.75	[0.947–8.007]
Black South African (ref non-Black South African)	1.17	[0.373–3.658]	1.49	[0.477–4.677]	0.83	[0.185–3.695]	2.03	[0.587–7.042]
Christian (ref non-Christian)	0.47	[0.113–1.958]	1.57	[0.430–5.709]	1.94	[0.468–8.006]	1.14	[0.243–5.347]
Time at facility (ref less than 5 years)								
5–10 years	0.71	[0.354–1.427]	0.7	[0.340–1.448]	0.29*	[0.118–0.717]	1.25	[0.535–2.897]
>10 years	0.92	[0.423–2.020]	0.79	[0.351–1.768]	0.4	[0.144–1.125]	0.88	[0.357–2.157]
Clinical Staff (ref non-clinical staff)	0.92	[0.534–1.593]	0.40*	[0.223–0.705]	0.73	[0.379–1.415]	0.32*	[0.159–0.625]

*p < 0.05.

**Table 3B:** Association of SABAS score and willingness to facilitate abortion care in scenarios 5–8 (*n* = 279).

	Scenario 5		Scenario 6		Scenario 7		Scenario 8	
	aOR	95% CI	aOR	95% CI	aOR	95% CI	aOR	95% CI
SABAS score	0.94*	[0.902–0.974]	0.92*	[0.876–0.977]	0.95*	[0.904–0.988]	0.93*	[0.899–0.969]
Female (ref male)	1.8	[0.853–3.820]	1.21	[0.409–3.550]	1.76	[0.749–4.110]	1.71	[0.803–3.654]
Age (ref <35)								
** **35–49	0.45	[0.181–1.135]	1.54	[0.436–5.418]	0.23*	[0.068–0.757]	0.38*	[0.154–0.951]
** **50+	0.45	[0.150–1.346]	1.54	[0.347–6.801]	0.33	[0.082–1.359]	0.49	[0.165–1.448]
Guateng (ref Limpopo)								
Marital Status (ref Single)	1.04	[0.570–1.897]	1.4	[0.594–3.313]	1.11	[0.558–2.216]	0.74	[0.407–1.332]
** **Married/living with partner	1.23	[0.624–2.415]	1.18	[0.460–3.019]	1.12	[0.517–2.415]	1.64	[0.836–3.203]
** **Divorced/Widowed/Separated	2.55	[0.898–7.235]	2.37	[0.543–10.332]	2.5	[0.710–8.780]	2.77*	[1.037–7.405]
Black South African (ref non-Black South African)	1.81	[0.519–6.338]	0.93	[0.098–8.939]	1.9	[0.450–8.059]	1.51	[0.437–5.206]
Christian (ref non-Christian)	0.57	[0.102–3.142]	0.92	[0.093–9.105]	0.61	[0.066–5.583]	0.48	[0.086–2.637]
Time at facility (ref less than 5 years)		** **	** **	** **	** **	** **	** **	** **
** **5–10 years	0.98	[0.431–2.223]	0.4	[0.103–1.523]	1.63	[0.636–4.194]	1.09	[0.482–2.461]
** >**10 years	0.95	[0.387–2.329]	0.29	[0.068–1.243]	1.09	[0.409–2.885]	0.67	[0.279–1.609]
Clinical Staff (ref non-clinical staff)	0.48*	[0.251–0.920]	0.87	[0.343–2.217]	0.8	[0.380–1.688]	0.44*	[0.232–0.843]

*p < 0.05.

For outcome measures 2 and 3 (actual facilitation or obstruction of abortion care), SABAS score was not significantly associated with facilitation of abortion care in the last 30 days (Table 4). In bivariate analyses, the mean SABAS score was 39.3 (range: 18–66) among those who facilitated access to abortion and 39.2 (range: 18–58) among those who did not (*p* = 0.946, data not shown). However, SABAS score was associated with obstruction of abortion care in the last 30 days; those who obstructed access to care had a mean SABAS score of 42.6 (range: 18–61), compared to 37.8 (range: 18–66) among those who did not obstruct access to care (*p* < 0.001, data not shown). This association held in adjusted analyses; with increasing SABAS scores, indicating higher levels of abortion stigma, health workers were more likely to obstruct access to abortion in the last 30 days (aOR 1.08; 95% CI 1.04–1.12).

**Table 4:** Association of SABAS score and facilitation or obstruction of abortion care in the last 30 days (*n* = 279).

	Facilitation in last 30 days		Obstruction in last 30 days	
	aOR	95% CI	aOR	95% CI
SABAS score	0.99	[0.944–1.049]	1.08*	[1.043–1.122]
Female (ref male)	0.97	[0.332–2.812]	1.59	[0.745–3.378]
Age (ref <35)				
** **35–49	1.37	[0.372–5.023]	0.55	[0.256–1.193]
** **50+	0.46	[0.110–1.911]	0.49	[0.186–1.274]
Guateng (ref Limpopo)	2.11	[0.861–5.173]	1.48	[0.840–2.613]
Marital Status (ref Single)				
** **Married/living with partner	0.55	[0.193–1.581]	1.39	[0.744–2.588]
** **Divorced/Widowed/Separated	1.01	[0.212–4.787]	0.5	[0.189–1.324]
Black South African (ref non-Black South African)	1.9	[0.404–8.894]	0.57	[0.177–1.857]
Christian (ref non-Christian)	2.25	[0.348–14.517]	1.48	[0.329–6.663]
Time at facility (ref less than 5 years)				
** **5–10 years	1.93	[0.578–6.462]	1.47	[0.697–3.099]
** >**10 years	1.62	[0.461–5.690]	1.16	[0.497–2.698]
Clinical Staff (ref non-clinical staff)	0.64	[0.251–1.649]	0.83	[0.458–1.501]

*p < 0.05.

**Figure 3:**
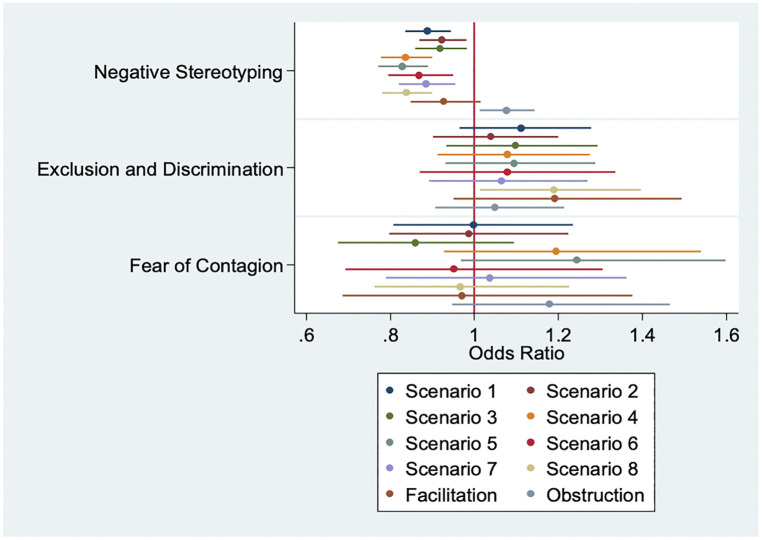
Subscale sensitivity analyses, all outcomes (*n* = 279).

In sensitivity analyses, the negative stereotyping SABAS subscale was shown to drive significance with our outcomes when controlling for socio-demographic characteristics; the negative stereotyping SABAS subscale was strongly inversely associated with every Scenario (Figure 3).

The other SABAS subscales were not associated across the eight scenarios; the exclusion and discrimination subscale was marginally significantly associated with willingness to facilitate access to abortion care in Scenario 8 (31-year-old requesting TOP due to non-use of condoms). However, this association operated in the opposite direction from that expected, with every one-point increase in exclusion and discrimination score significantly increasing the odds of being willing to facilitate abortion care in Scenario 8 (Figure 3; aOR 1.19 95% CI: 1.01–1.40). The negative stereotyping subscale was similarly significantly associated with the obstruction outcome (aOR 1.08 95% CI: 1.01–1.14); no other subscales were significantly associated with this outcome. As with the overall SABAS score, no SABAS subscales were significantly associated with the facilitation outcome.

## Discussion

Overall, this sample of clinical and non-clinical health facility workers in Gauteng and Limpopo provinces of South Africa had low to medium levels of stigmatizing attitudes, beliefs, and actions towards women seeking an abortion, with significantly higher levels of stigma among non-clinical staff when compared to clinical staff. This finding echoes existing literature that abortion stigma and obstruction is often experienced at all levels of health facility staff and is not limited to health care workers directly providing the service (16). We found that respondents are largely willing to facilitate access to abortion care both in theory (the eight hypothetical scenarios) and in practice (facilitation). Over 50% of respondents in our sample were willing to facilitate abortion care in each of the eight scenarios, and over 90% reported facilitating abortion care in the last 30 days. However, one in three respondents (31%) reported having done something to obstruct abortion care in the last 30 days. We anticipate these numbers may be higher, given the possibility of underreporting obstructive behaviors. These data also make clear that some health facility staff are both facilitating *and* obstructing abortion care, adding to the evidence base the nuance of provider decisions around participation in abortion care (14, 16–18) and facility staff power to influence health outcomes.

Our results also show clear divisions among health facility staff in willingness to facilitate abortion care across scenarios, with highest willingness to facilitate in Scenario 6 (congenital defects) and 7 (rape) and lowest willingness to facilitate in scenarios for which gestational age was specified as after 13 weeks (Scenarios 1 and 2). Scenario 3 (cervical cancer) also specified gestation beyond 13 weeks, but 78% of our sample expressed willingness to facilitate abortion, which demonstrates a discomfort with abortion facilitation beyond the first trimester for reasons other than life, physical health, or congenital defects, in line with existing literature (14, 23–25). These results again highlight the power of health facility staff in gatekeeping access to facility-based abortion care. Respondents demonstrate more openness towards first-trimester abortion and abortion in certain situations (like rape and congenital defects) that are perhaps seen as more sympathetic or out of the woman's control, when compared to reasons like staying in school, keeping a job, or contraceptive failure. In other words, the health facility staff in our sample are willing to facilitate abortion care for some and obstruct it for others—the very definition of discrimination. People who face discrimination and denial of abortion services are more likely to carry unwanted pregnancies to term, affecting the health and well-being of women and their families (26).

Our results also show a clear association between stigma and willingness to facilitate access to abortion care. SABAS scores were significantly inversely associated with all of our Scenario outcomes when controlling for covariates, demonstrating that higher levels of stigma are associated with lower odds of willingness to facilitate access to abortion care. No other variables were found to be significantly associated with our outcomes across all eight scenarios. Interestingly, despite lower average SABAS scores in general, clinical staff were found to be less likely to facilitate abortion care across all scenarios when compared to non-clinical staff and when controlling for covariates; this association was significant in four scenarios (Scenario 2, 4, 5, and 8). This may be due to the fact that facilitation of abortion care may hold a different meaning for clinical staff than it does for non-clinical staff, with facilitation translating into direct provision of the abortion service for clinical staff.

While stigma was associated with hypothetical or theoretical willingness to facilitate abortion care, this association did not hold for *actual facilitation* of an abortion service in the last 30 days, illustrating important differences between facilitation of abortion care in theory and in practice. Clearly, while health facility staff may be theoretically willing to facilitate abortion care, the lived practice of this does not align, likely due to an individual's assessment of the situation—and the woman seeking the abortion—in the moment, again pointing to discrimination. Conversely, stigma was associated with *actual obstruction* of an abortion service in the last 30 days, with higher SABAS scores (higher levels of stigma) associated with higher odds of obstructing abortion care. This suggests that stigma plays a larger role in obstruction than facilitation in this setting, which is striking given that South Africa's CTOP Act makes it illegal for anyone to prevent a lawful TOP service or obstruct access to a facility for this purpose (4).

When we run the models using the SABAS subscales, associations between stigma and our outcomes were shown to be driven largely by the negative stereotyping subscale—those statements that see women who get abortions as sinful, shameful, untrustworthy, and bad influences. These findings, coupled with the fact that obstructive behavior occurred among clinical *and* non-clinical staff, point to a need to work toward reducing stigma among *all* health workers. Our analysis specifically identifies negative stereotypes against women receiving abortion as key to increasing willingness to facilitate abortion care and reducing obstruction of abortion care. Furthermore, the non-significance of interaction terms between Christianity and SABAS score suggests that, in South Africa, stigma's impact on abortion services may not be mediated by religion. While this finding may differ in other settings where religious institutions are more heavily involved in anti-abortion rhetoric, it is important to acknowledge that stigma against women receiving abortions is cross-cutting and not unique to those who practice religion.

This study has several limitations. This study was conducted using baseline data from 279 clinical and non-clinical staff in 16 health facilities in Gauteng and Limpopo provinces, all of which have at least one Ipas-trained abortion provider. As such, these health facilities may not be representative of all facilities where abortions are offered in South Africa and may have lower overall levels of stigma when compared to other health facilities. We relied on hospital administrators to define levels of engagement with abortion clients in an effort to sample only those who have medium to high levels of engagement with abortion service. These administrators know their staff's responsibilities best; however, it is possible some respondents had little to no interaction with the abortion service, thus skewing results on facilitation and obstruction towards the null. Finally, surveys were interviewer-administered by Ipas staff and consultants. The staff and consultants were not known to study participants, but the Ipas affiliation may have contributed to social desirability bias in outcomes of interest and stigma measures, and particularly in reporting obstruction of an abortion service in the last 30 days. We expect any bias to be minimal as Ipas was primarily known to abortion providers who received training, and these providers were not included in the present analysis. In addition, confidentiality of data, administration of surveys in private areas, and training of interviewers were emphasized in an effort to diminish possible social desirability bias.

## Conclusions

Stigmatizing attitudes, beliefs, and actions toward women who seek abortion is crucially important in shaping how clinical and non-clinical staff in South Africa feel about their participation in abortion care and, ultimately, whether or not they obstruct this care. Facility staff hold great power in determining whose abortions are facilitated and whose are obstructed, resulting in stigma and discrimination being openly perpetuated. Efforts to reduce stigma towards women seeking abortion, and particularly negative stereotyping, among *all* health facility staff is key to ensuring equitable and non-discriminatory access to abortion for all.

## Data Availability

The raw data supporting the conclusions of this article will be made available by the authors, without undue reservation.

## Footnotes

^1^While not all people who access abortion identify as women, we use the term “women” in this article as abortion stigma is also particularly gendered (7). “Women” also reflects the terminology used in the baseline surveys under analysis in this research.
