# Supplementary material for: Stigma towards women requesting abortion and association with health facility staff facilitation and obstruction of abortion care in South Africa
Source: Front Glob Womens Health. 2023 Jun 15;4:1142638. doi: 10.3389/fgwh.2023.1142638 (PMC10311091; doi:10.3389/fgwh.2023.1142638)
Supplement: Supplementary file 1 [file Table1.docx]

**SUPPLEMENTAL FILE 1: Stigmatizing Attitudes, Beliefs and Actions Scale (SABAS)**

|  | ***Negative Stereotyping*** |
| --- | --- |
| 1 | A woman who has abortion is committing a sin |
| 2 | Once a woman has one abortion, she will make it a habit |
| 3 | A woman who has had an abortion cannot be trusted |
| 4 | A woman who has an abortion brings shame to her family |
| 5 | The health of a woman who has an abortion is never as good as it was before the abortion |
| 6 | A woman who has had an abortion might encouraged other women to get abortions |
| 7 | A woman who has an abortion is a bad mother |
| 8 | A woman who has an abortion brings shame to her community |
|  | ***Exclusion and discrimination*** |
| 9 | A woman who has had an abortion should be prohibited from going to religious services |
| 10 | I would tease a woman who has had an abortion so that she will be ashamed about her decision |
| 11 | I would try to disgrace a woman in my community if I found out she’d had an abortion |
| 12 | A man should not marry a woman who has had an abortion because she may not be able to bear children |
| 13 | I would stop being friends with someone if I found out that she had an abortion |
| 14 | I would point my fingers at a woman who had an abortion so that other people would know what she has done |
| 15 | A woman who has an abortion should be treated the same as everyone else |
|  | ***Fear of contagion*** |
| 16 | A woman who has an abortion can make other people fall ill or get sick |
| 17 | A woman who has an abortion should be isolated from other people in the community for at least 1 month after having an abortion |
| 18 | If a man has sex with a woman who has had an abortion, he will become infected with a disease |

***Note:*** All statements answered using the following 5-point Likert scale: Strongly Disagree, Disagree, Unsure, Agree, Strongly Agree
